# Intergenerational Theater Workshops as Unique Recreational Activities among Older Adults in Japanese Care Facilities: A Qualitatively Driven Mixed-Methods Multiple-Case Study Design

**DOI:** 10.3390/ijerph191811474

**Published:** 2022-09-12

**Authors:** Michiko Abe, Ren Gyo, Junro Shibata, Kentaro Okazaki, Rumiko Inoue, Tatsuki Oishi, Machiko Inoue

**Affiliations:** 1 Department of Family and Community Medicine, Hamamatsu University School of Medicine, Hamamatsu 431-3192, Japan; 2 Graduate School of Management, Kyoto University, Kyoto 606-8501, Japan; 3 Graduate School of Core Ethics and Frontier Sciences, Ritsumeikan University, Kyoto 603-8577, Japan; 4 Community Medicine Education Unit, Faculty of Medical Sciences, Kyushu University, Fukuoka 812-8582, Japan; 5 Care Styles Consulting, Osaka 541-0048, Japan; 6 Fringe Theatre Association, Kyoto 600-8445, Japan

**Keywords:** intergenerational activity, well-being, art-based recreation, theater, care settings

## Abstract

Intergenerational theater activities have been recently employed in recreation for older adults. We held a series of four intergenerational theater workshops in two older adults’ care facilities in Japan and sought the experiences of older participants, younger participants, and the facility managers. With a qualitatively driven mixed-methods multiple-case study design, we obtained data from field observation, interviews with participants, and preworkshop and postworkshop changes on a well-being scale (Ikigai-9) among older participants, and the results of the two sites were compared. “Immediate effects” were seen in older adults because they responded actively and demonstrated surprising faculties during the workshop. Facility staff members and younger participants received “extended effects” because they gained new ideas regarding the remaining skills of older participants and a sense of reuniting with old neighbors through the exercise. In the Ikigai-9 scale, the items measuring “present happiness” significantly improved at Site 1 but not at Site 2. Better results at Site 1 might have been caused by the lower care needs of participants and the inclusion of children. Less support from facility staff members during the activities also might have promoted the voluntary participation of older adults. Involving children and engaging the facility staff in preparation could enhance the quality of activities.

## 1. Introduction

Maintaining and promoting the well-being of older adults in long-term care is a global concern in the current aging society [1,2,3]. According to the Centers for Disease Control and Prevention (CDC), the definition of well-being includes “the presence of positive emotions and moods, absence of negative emotions, satisfaction with life, fulfillment, and positive functioning [4].” It is an important goal for people under long-term care because they live with some level of physical or mental disability and feelings of loneliness by being away from home [1,5]. They likely restrain their desires and lose a sense of self-control over their lives by increasingly depending on others instead of gaining physical security in the facilities [6]. Therefore, for better health and well-being, older adults need contributing roles that will motivate them to participate in meaningful activities [2,7], offer the chance to learn new skills to increase independence, and bring a sense of being a part of the community [5]. Such care approaches require collaborative efforts with caregivers, younger generations, and many sectors of the community [2]. Thus, the kind of activities and ways in which we can improve the well-being of older adults in long-term care is a crucial research topic [2].

There is a growing interest in the effect of arts on the well-being of older adults [8,9,10]. Art-based activities, such as music, dance, and theater, are becoming more recognized as relevant tools to enhance participants’ physical movement, communication skills, and memory through group activities [8]. Such activities are usually arranged for older adults to work not only with artists but also with caregivers, neighbors, and children, instigating an intergenerational exchange [11,12]. The current studies of intergenerational art programs have shown positive effects on the well-being of older adults [9,13], in addition to younger generations receiving inspiration from older adults and reducing fear of growing older [14] and promoting mutual understanding between the young and old [9,11,15]. Therefore, incorporating an intergenerational approach in art-based activities has become appreciated as an opportunity for social participation for older adults [9,10,13,16].

Concurrently, the inclusion of theater is a novel idea among the various forms of art-based recreation in care settings [17,18]. Hence, the programs that examined the impact of the theatrical activity on older adults with care needs include the Storybox Project [19], Hearts and Mind [20], and the TimeSlips project [21]. In these programs, under the facilitation of theater experts, participants enjoy creating plays in a failure-free context [21,22]. Bernard and Rickett’s (2017) review states that the values of theater participation for older people are improvement in well-being, group relations, and learning and creativity [17]. However, the studies focusing on the intergenerational aspects of theater are limited [13,14]. Most studies of theater-based activities in care settings have been conducted in Europe and North America, and reports from Asian countries are minimal. Older adults have not been analyzed enough through the lens of dynamic relationships between the findings and practice resources in previous studies on health and creative programs [23].

In Japan, with the highest aging population in the world, about 30% of the population aged 75 and older, i.e., approximately five million older adults, currently receive various care services through government long-term care insurance [24]. Recreational activities for the aged are mostly music, games, arts and crafts, outdoor activities, and nature exploration [25]. Although older adults’ theater activities in Japan have been identified since the 2000s, they mainly involve active older people, not those with care needs [26,27,28]. In our previous study, the authors developed and implemented intergenerational theater workshops for active older citizens and working-age people. It was a series of four workshops facilitated by trained actors and about 20 participants in their 20s to 80s. We found positive results among the participants, which included an improved sense of closeness, respect, and gratitude among intergenerational participants [29]. Based on the positive results and by learning from other research in Europe and North America, we considered applying this theatrical approach to the Japanese care settings and examining the impact of the program in terms of promoting older adults’ well-being and social participation.

This study aims to explore the impact of our intergenerational theater workshops in Japanese older adult care facilities in a multiple-case study design. Our research questions are as follows: (1) How will the intergenerational theater workshop affect the participants: older adults, younger participants, and facility staff members? (2) Will the workshop improve older participants’ well-being? (3) Under what circumstances would it be more effective for older participants’ well-being? To add value to previous studies, we would explore the environmental factors [23] that could help improve the effectiveness of the workshops by comparing practices at two sites.

## 2. Materials and Methods

### 2.1. Study Design

We used a mixed-methods multiple-case study design [30,31] based on the paradigm of pragmatism, which allows for a plurality of views and methods to be a part of its overall research plan [32]. A case study is a research design involving an intensive and holistic examination of a contemporary phenomenon in a real-life setting [30]. We chose this design to capture the context-dependent and interactive nature of this study’s target activity. 

Employing mixed methods further allowed us to understand the impact of workshops in a multidimensional approach from the perspectives of various stakeholders through merging both qualitative and quantitative data. For qualitative data, we used field observation and video recordings of all workshops, as well as interviews with facility managers and younger participants. Further, we gathered short comments from older participants at the end of the final session. To quantitatively assess the preworkshop and postworkshop changes in the older participants’ subjective sense of well-being (“ikigai” in Japanese), we administered the Ikigai-9 scale [33,34] before the first session and right after the fourth session. This scale was used because it consists of nine short questions with plain wording (9-item questionnaires), and we considered it applicable for participants with mild cognitive decline, as well as those with normal cognitive functions. 

**Figure 1:**
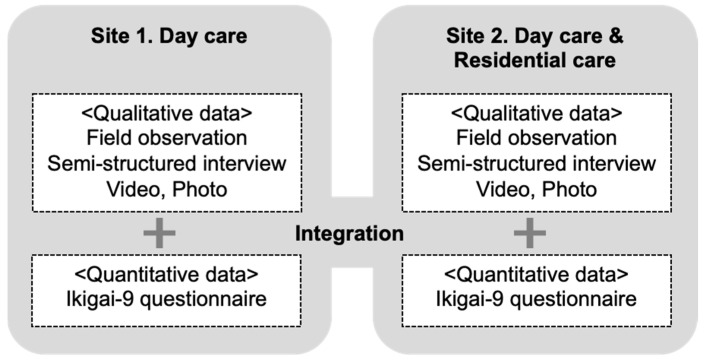
A qualitatively driven mixed-methods multiple-case study design. The qualitative data analysis was conducted, and the quantitative data were then examined. The data set of each site were compared to explore the elements of the implementation contributing to the well-being of the older participants.

We initially conducted qualitative data analysis to extract the observations of the impact of the activities and the characteristics of each site (Research question 1). We then examined the quantitative results of the well-being questionnaire of each site and explored the elements of the implementation that could contribute to the well-being of older participants by comparing the two cases (Research question 2). We included this quantitative measure to gain supportive data to enable comparison between the two cases even with a small amount of data in each case. Last, we derived further understandings of the elements of workshops that can be effective, considering qualitative data (Research question 3). The design of this study is a “qualitatively driven” mixed-methods multiple-case study since the quantitative evaluation was used as a supplementary tool to deepen our understanding of qualitative results. Figure 1 presents the study’s design structure.

### 2.2. Description of the Theater Workshop and the Provider

This theater workshop was designed and produced by the Fringe Theater Association, a Kyoto-based nonprofit organization founded in 2018. They work on theatrical projects for youth experiential learning and recreation in care facilities, with a current roster of approximately 20 facilitators. They employ improvisational theater techniques with the basic spirit of “Yes, and”, which means they observe and sense what is happening in the place and always welcome ideas from participants and add them to their creation positively [35] (p. 59–71). The emphasis is on enjoying the “process” of creating theater as a group rather than completing an artistic work.

The program was a series of four one-hour workshops, as presented in Table 1. Three trained actors led a group of 10 to 15 intergenerational participants. The workshop began with greetings and communication games. This initial activity functioned as a guide for participants to be part of the improvisational theater, and learning any type of reaction was highly appreciated in the workshop. The facilitators introduced a basic storyline from the theater at the end of the first session. They took the basic story from a folk tale familiar to all generations and continued to add or change elements of the story by adopting participants’ reactions throughout the sessions. In the second session, facilitators cast their participants. Older participants were divided into several groups to share a role, and a facilitator and some younger participants were assigned to support each group. Participants practiced the scenes led by facilitators through the second and third sessions. Scripts for the play were shared only among the facilitators who directed each scene. While some key lines were given to the younger participants, older participants did not have to memorize any lines. Instead, facilitators asked them specific questions in several scenes to elicit their reactions in a natural flow. Improvisation was welcome at any point in the play. In the final session, the participants presented the play in front of an audience consisting of facility users who did not participate in the workshop, and staff members. The play was closed with a song conversant to all workshop participants and that the audience could sing together.

### 2.3. Selection of the Study Site and Participant Recruitment

Because most care facilities in Japan are not familiar with theater workshops, we took a strategy of chain and opportunity sampling [36] (pp. 270–271) for selecting study sites and participants. We approached facilities already connected to the Fringe Theater Association and asked if they were interested in participating in the study. Site 1 was a daycare center that had conducted a similar workshop the year before. Site 2 was a daycare and residential care facility without any experience hosting a theater workshop but accommodated us through a personal connection with one of the facilitators. We provided a 500 yen gift card (equivalent to USD 5) to the participants of the workshops and facility managers who answered our interview.

Older participants were recruited in advance at each care facility by caring staff who asked if they would like to participate in “play-making with actors.” Staff gently advised the facility users that they were free to leave the room after it began if they did not like it, for those who could not imagine what they were being called to do. For research purposes, we asked the care facility manager to provide a list of planned participants with information on their age, gender, a certified level of care needs based on the governmental system of long-term care insurance, and frequency of facility use.

Neighborhood residents were recruited through local newsletters and social networking systems for Site 1. For Site 2, the local town coordinator invited amateur theater group members. We asked the facility care staff to help with delivering instructions to those with hearing issues and assist those who looked tired or needed a restroom. The Fringe Theatre Association appointed three facilitators for each site, one as the lead facilitator and the others in a supporting role. They were selected for trusted facilitation skills, and the team of three for each site was arranged by combining those with more and less experienced in working with older adults in care settings.

### 2.4. Data Collection Instrument and the Procedure

#### 2.4.1. Field Observation of the Workshop

All workshops were videotaped, photographed, and observed by the research team. Field observation was based on the ethnographic tradition by using the format of the 3Cs summary [37] to describe the context, contents, and concepts. Two or more researchers were assigned for each session to a corner of the room where they would not interfere with the activity. One of them observed the lead facilitator, and the others focused on several participants and wrote descriptive observations during the workshop. Observers also conducted informal interviews with neighborhood participants after the workshop, asking them about their impressions of the activity.

#### 2.4.2. Semi-Structured Interview of Care Provider

We interviewed facility managers from each site after the final workshop. The interviews were semi-structured with a guide developed by the research team. The questions included the following: “What kind of memorable changes did you see in the older participants?”, “Did you find the workshop effective as a rehabilitation?”, “Did the activity provide any change to the perspective of facility staff?”, and “What were the advantages and disadvantages of this workshop?” The interviewers were the research team members who conducted field observation.

#### 2.4.3. Pre–Post Changes on the Well-Being Survey Using Ikigai-9 Scales for Older Participants

Older participants were given the well-being questionnaire using the Ikigai-9 scale [33,34] before the first session and right after the fourth session. Ikigai-9 is a psychometric tool tested for reliability (Cronbach’s alpha = 0.87) and validity for the use with the older Japanese people. The scale included three subscales: “optimistic and positive emotions toward life”, “active and positive attitudes toward one’s future”, and “acknowledgment of the meaning of one’s existence.” Participants responded to nine questions on a five-point Likert scale. The research staff assisted each participant with the questionnaire by presenting the questions legibly, in large letters, reading them aloud, and asking them to select their answers. 

We added the following question at the end of the post-workshop survey, “What was your impression of the theater-making activity?” and wrote down older participants’ comments in case of any reactions. These comments were used for the qualitative data analysis. 

#### 2.4.4. Ethical Considerations

We informed the participants regarding the voluntary nature of study participation and their freedom to withdraw from the study. For the older people who could not give informed consent, we asked facility staff to confer with their family members or adult guardians for approval. The study protocol was approved by the Institutional Review Board of Hamamatsu University School of Medicine in Japan (No. 18-273) and was registered at the UMIN Clinical Trials Registry (UMIN000036617).

#### 2.4.5. Data Analysis

The findings from field observation were written in the form of a 3Cs summary [37] by our researchers. Semi-structured interviews with facility managers and neighborhood participants were recorded and transcribed verbatim. We analyzed the data using thematic analysis, a “method for identifying, analyzing and reporting patterns (themes) within data” [38]. The main analysts (J.S. and M.A.) read through individual data for possible codes. Codes were then collated into documents, titled by theme, consisting of excerpts of raw data and their corresponding analyses. Video recordings were reviewed when analysts needed to validate the actual happenings when analyzing the descriptions in 3Cs summary. The concepts were shared digitally within the team and discussed in team meetings. 

The well-being scale Ikigai-9 [33,34] collected from older participants was analyzed using the Wilcoxon signed-rank test for the significance between the pre- and post-workshop. We first compared the Ikigai-9 results site by site and then cross-checked them with the qualitative analysis to consider the factors influencing the differences in Ikigai-9 outcomes, such as the participants’ demographics and implementation context. Finally, the characteristics of each site, Ikigai-9 results, and our meta-inferences were summarized.

## 3. Results

### 3.1. Description of the Site and Study Participants

The settings of each site are described below. Table 2 presents the detailed characteristics of the older participants with the distribution of physical and cognitive conditions.

Site 1 was a daycare center. We held workshops on Saturdays for four consecutive weeks in June 2019. Because this facility experienced similar workshops the previous year, the preparation process went smoothly with the manager, and some of the older participants were familiar with the workshop facilitators. Eleven older individuals signed up for the workshop, with an average age of 86. Most of them were certified at care level 1 or 2, with difficulties performing essential daily living tasks. The younger participants included three to four adults in their 30s and 40s who became aware of the workshop by the call for participation through social networks. Four of the research team members’ children and their friends (aged 3–11 years) also joined the sessions. The facility manager remained in the room to offer workshop support, but other staff members came in and out, occasionally checking in on the situation as they tended to other tasks. The venue size was relatively small for the number of participants. The fourth workshop was held in the facility’s dining room so most staff members could watch the performance.

Site 2 was a combined daycare and residential facility. We held workshops on two separate weeks (Fridays and Saturdays) in September 2019. Because this facility had never experienced a theater workshop, the manager was anxious regarding whether the participants could enjoy the activity and how they should prepare for the event. Sixteen participants signed up for the workshop, with an average age of 87. Half were daycare users, and the others were residents. This group’s certified care levels were broader than those of Site 1, from independent to care level 4, meaning “faces difficulty living without constant care.” Three to eleven volunteers from a local amateur theater group participated along with the staff from a public consumer center using theater to help in protecting older citizens from crimes. Two to four facility staff attended the workshop site because the residential facility allowed them to concentrate on the recreation activity and because some of the older participants required a high level of care. As the staff became more familiar with the workshop, they enthusiastically supported the participation by anticipating the facilitator’s intentions and conveying detailed instructions to the ears of the participants.

### 3.2. Research Question 1: How Will the Intergenerational Theater Workshop Affect the Participants? 

The Theater Together Project provided the older participants with enjoyment and stimulation by working with visitors to create a play. At the beginning of the first workshop, a sense of tension was noted among the participants because they wondered what would happen subsequently. Because the workshop proceeded step-by-step from one type of communication game to another, the older participants gradually learned how to enjoy the activity. We were particularly interested in the impact of working as an intergenerational group and were able to observe the group dynamics and interaction among the older participants or facility users and neighborhood residents. For example, at Site 1, four children (aged 3–11 years) attracted the older participants and adults, which helped create a sense of unity among them. In a communication game, wherein participants sat in a circle and passed imaginary items around to the next person, the three-year-old child was initially too shy. The older participants and adults paid attention to such children’s reactions, cheering them on, and rejoicing with them when they did well, which increased the cooperation of the whole group. Notably, the older participants, who are usually in the role of “receiving care” in the facility setting, seemed to try managing themselves in front of the children. 

The second example was at Site 2, located far from the facilitator’s operation base. When an older participant spoke a dialect in the communication game, a local adult participant translated it to the facilitator, which gave the impression that the facilitators and the rest of the participants were residents of different cultures. The facilitator then inserted a scene in the play wherein neighborhood participants introduced their town to attract travelers. The 97-year-old woman, who had previously shown only a weak reaction to the activity, laughed and nodded in response to the descriptions of the local scenery in their dialect. We observed a cultural bond between the older participants and neighborhood participants in such reactions. The different backgrounds of the participants could create a contrast between uncertainty and familiarity in the group activity, and the difference was utilized as social stimulation for the older participants.

**Figure 2:**
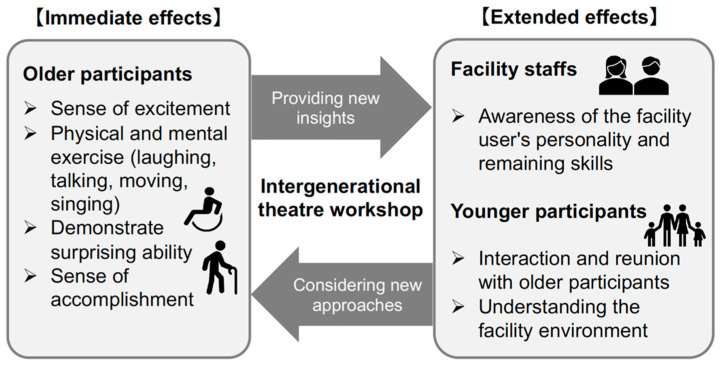
The impact of the intergenerational theater workshop. Themes overview of the impact of the intergenerational theater workshop emerged from the qualitative analysis.

Through the thematic analysis of field observation and the interviews, two qualitatively different themes of the impact of the intergenerational theater workshop emerged. One was the “immediate effects” for the older participants, and the other was the “extended effects” for the facility staff and younger participants. Figure 2 presents the theme overview, and the details are described in the following sections.

#### 3.2.1. Immediate Effects

An “immediate effect” was observed in the older participants’ behaviors and reactions during the workshop. Their active responses included laughing and applauding others’ actions, smoother movements in the game, improvised conversations or movements, trying to help others participate in the game and scenes, and singing aloud. At the end of the final performance in front of the audience, there was a sense of accomplishment in their expressions.

Thirteen of the older participants left comments after the final session, although they were very brief considering their fatigue after the activity. The expressions that emerged several times were “worked with everyone”, “participated in the play”, and “it was fun,” thereby indicating that many older participants obtained a sense of enjoyment from doing theater workshops as a group. At Site 1, two persons commented that they were impressed by the children’s performance. However, two participants commented that “I didn’t understand what was going on” and “I couldn’t hear some things,” which suggests that they needed more individual support.

The “activeness” of older participants was highly appreciated by the facility managers, who knew their usual conditions. The manager of Site 1 mentioned, “The excitement of the participants as a group was much greater than I expected compared to the usual recreational activities.” The manager of Site 2 said, “Thanks to the various activities presented by the facilitators, our users who usually do not express themselves were processing some level of thinking during the activity.” They both observed different reactions from the older participants than their usual comportment in recreation activities.

When we asked regarding the rehabilitation effect of the workshop, the managers valued the activity as “good for the users’ mental health rather than physical impact” (manager, Site 1). Another said, “The older participants may not always remember the activity, and it could have been a fresh amusement for them each time” (manager, Site 2). In contrast to the traditional rehabilitation in care facilities, focused on improving or maintaining the physical function of individuals, the intergenerational theater workshops demonstrated success in elevating emotional fulfillment in the older participants.

#### 3.2.2. Extended Effects

In addition to the “immediate effects” observed in older participants in the workshops, the people around them also received benefits that could potentially continue after implementation. The facility managers gained new insight into the personality and remaining faculties of the older participants. Comments included that “I have been maintaining my distance from the quiet persons not to offend them by trying to say something funny. However, it was good to learn that they would react to the jokes in such playful attitudes.” (facility manager, Site 1) and “I didn’t know she could do such a thing, maybe I’ll ask her to cook some time” (facility manager, Site 2), particularly for the older participant who repeatedly presented her cooking recipe in the play. These findings could significantly add new options to their daily care routines and interactions.

The younger generation also appreciated the workshop opportunity. The adult participants at both sites were somewhat familiar with theatrical activities and often supported the facilitators. They helped in passing along instructions to older participants and took charge of key lines in the play. In an interview after the workshop at Site 2, one younger participant commented on an older participant who used to be her neighbor saying, “In the beginning, she seemed to be less talkative and pessimistic, but as the workshop went on, she regained her former cheerful personality” (adult participant, Site 2). While observing the “immediate effects” of the workshop on older participants, the adult participants admitted that the workshops became an opportunity to reunite with old neighbors and discovered that their regular visits could help in improving an old friend’s well-being. In the case of the children at Site 1, at the end of the last session, one of them asked, “Can’t I see everyone anymore?” indicating that they became friends with the older participants through the sessions.

Furthermore, an adult participant stated, “Once they are in a facility, we have no idea what kind of life they lead from the outside,” (adult participants, Site 2) suggesting that the theater workshops in care facilities could promote beneficial exchanges and mutual understanding among neighborhood residents and facility users.

### 3.3. Research Question 2: Will the Workshop Improve Older Participants’ Well-Being? 

The Ikigai-9 [33,34] questionnaire, a scale of well-being for older adults, was given to the older participants before and after the workshop series. The results are presented in Table 3. The older participants who responded both in the preworkshop and postworkshop questionnaires were limited to 10 at site 1 and 8 at site 2 because some registered participants were missing in the first or the fourth session. Although the distribution of age and care needs was similar to the overall number of registered older participants, we needed to interpret the quantitative results with caution because of the small number of respondents.

In Site 1, among the three subscales, the results of “I. Optimistic and positive emotions toward life” and question (5) under subscale II, “I am interested in many things,” were significantly improved. These items were questions on present happiness and fulfillment. Other questions that measured feelings regarding the future or regarding one’s relationship with society did not significantly change. The Ikigai-9 results from Site 1 correspond with our observations of the “immediate effects” of the workshop on older participants.

On the other hand, in Site 2, we did not find significant improvements on any questions. One of the reasons was the prescores for the items with significant changes in Site 1 were initially higher in Site 2. Additionally, more participants in Site 2 required a higher degree of nursing care and had cognitive decline, so it could have been more difficult for the older participants to understand the conceptional nature of the questions on the scale. Therefore, the results of Ikigai-9 should be carefully compared with the qualitative observational data, as discussed in the next section.

### 3.4. Research Question 3: Under What Circumstances Would It Be More Effective for Older Participants’ Well-Being?

Given the gap between the results of Ikigai-9 at Sites 1 and 2, we analyzed the differences in the environment where the workshops were held that could influence these results (Table 3). First, Site 2 was a combined daycare–residential facility, so the range of care needs among the older participants was broader than that at Site 1, and some required higher-level care (Level 3 to 4 in Table 2). We assume that it is more challenging to satisfy everyone with such a variation in participants’ cognitive and physical conditions. The facilitators may need to develop the skills for this environment.

Second, Site 1 was more advantageous than Site 2 because they were more familiar with theater and had conducted a similar theater workshop a year before with the same facilitators. By contrast, this was the first time Site 2 held such a workshop. We observed that it required more effort in Site 2, especially in the earlier stages, to communicate with older participants about how to play the games compared to Site 1. There is potential to improve the quality of implementation if facilitators, facility staff, and participants gain some degree of experience with this activity and create a more relaxed atmosphere from the start.

Third, regarding the younger participant attributes, four children were included in Site 1, and in Site 2, there were only adults and no children. In Site 1, children’s contributions were energizing and significantly engaged the older participants in the workshop. It may have influenced the positive results of the “present” well-being measured in a subscale of Ikigai-9. We observed that the adult participants from the neighborhood also added value to the workshops. Their feedback had the potential to positively affect the third subscale of Ikigai-9, “Acknowledgment of the meaning of one’s existence (in a society).” However, compared to the children’s direct impact, this aspect may take some time to be appreciated by older participants. Here again, we suggest that continuity of the program is a key strategy for intergenerational theater workshops in a care setting.

Fourth, we considered why the “present” items of the older participants’ Ikigai-9 scale increased in Site 1, despite less support from the facility staff. It is possible that less staff intervention meant more direct interaction with visitors who had no biases regarding the older participants. The older participants may have found a sense of accomplishment participating in an extraordinary event. The enthusiastic support of the facility staff at Site 2 may have limited the space for older participants to display their abilities. To make the best use of the theater workshop run by outside human resources, we may need to explore a balance between supporting and waiting for older participants’ reactions.

## 4. Discussion

This study examined the impact of our unique program, the Theater Together Project, an intergenerational theater activity in care facilities for older adults through participant observation, interviews, and the Ikigai-9 survey on well-being [33,34]. The qualitative results suggested that the positive effect of the workshops on the reaction of older participants and the views of younger participants and facility staff. However, in the well-being survey for older participants, items that showed significant improvement were limited to present well-being at site 1. The gap in the results between the two sites offered valuable insight into environmental factors that could contribute to the well-being of older participants and areas for improvement in our activity: facilitators’ skill development for a group with a broad range of care needs, continuity of the activity, the inclusion of children, and facility staff to refrain from supporting older adults during the activity so that they can directly interact with visitors. The dynamic inference of the activity’s effect and the environment was a unique contribution of the present study, while previous research on older adults’ creative activities has focused mainly on the effects of implementation with outcome-driven research questions [11,23].

Our observations and interviews found the significance of implementing theater-based activity for the well-being of older adults in care settings. The activity offered them a new role to play, a story to contribute, and a purpose to perform for the audience. The definition of well-being was “the presence of positive emotions and mood, absence of negative emotions, satisfaction with life, fulfillment, and positive functioning [4]. When considering that current recreational activities in Japanese care facilities focus on maintaining individuals’ physical functions [39,40], the Theater Together Project demonstrates different attributes to stimulate the mental and social aspects of life for older people. Swinnen and De Medeiros (2017) found the meaning of “play” in participatory art programs in dementia care settings: it gives people emotional connection and the freedom to express oneself [41]. Harries (2013) conducted creative activities including drama for older people with dementia and reported that “the participants try new things and push the boundaries of what people typically assumed they were capable of [19].” The present research in Japan supports those benefits reported in previous studies. Therefore, those interested in applying theatrical methods to improve the social well-being of the older generation in Japan [26,28,42,43] and care facilities that seek creative activities to promote the well-being of their users can turn to this study for confirmation [25,44].

Regarding the intergenerational aspect of the activity, one element that could positively affect the present well-being score of older participants is the presence of children. At Site 1, where children participated, older participants paid particular attention to the children and showed a willingness to care for them. This finding is a result similar to that of Gaspar (2022) concerning intergenerational theater production with children and youth participants, in which older participants found a sense of satisfaction, purpose, and accomplishment, as well as something to look forward to [13]. The review of Gualano et al. (2018) on intergenerational programs for older adults and children, although theater programs were not included, reports that most studies agreed concerning the positive effect on the psychosocial well-being of older participants [12]. The emotion stimulated by interactions with children might be difficult for adult caregivers or visitors to elicit from older people. Therefore, when planning intergenerational theater workshops, collaborating with nearby schools or encouraging neighborhood families to bring children to the workshops is worth consideration [13]. 

However, we need to note the other benefits to the presence of intergenerational participants aside from the role of children. At Site 2, where the age range of younger participants was from the 40s to 70s, we observed that shared culture in their community, such as beautiful scenery or seasonal changes, were described and appreciated in the play. The intergenerational participants understood the significance of periodic visits from the reactions of their old friends welcoming them. Gasper et al. (2022) compared the impact of intergenerational theater activity on the children’s group (ages 5–11) and youth group (ages 12–17). They report the interaction with older adults as having more meaning for youth participants, allowing them to explore various roles and ideas with exposure to various age groups [13]. Anderson (2017) studied intergenerational theater programs with older adults and university students (ages 21–62) and found shared well-being outcomes from both generations, such as reduced agism/fear of aging, increased social networks, enhanced self-confidence and self-esteem, and increased empathy and equanimity [14]. Dassa & Harel (2020) worked with performing art school students (ages 23–27) and older adults with dementia and found that the mutual engagement in a creative process made the students honor and admire the person with dementia, which enabled them to see beyond the disease [16]. Together with the knowledge from those previous studies, it is suggested that including mature younger participants can have a deeper meaning of fostering mutual understanding on both generations.

Another finding from this study was that collaboration between workshop facilitators and facility staff needs careful planning to enhance the activity’s effectiveness. Throughout the study, facility managers emphasized the value of this initiative in care settings. Because older participants entertained facility staff members in their performances and the staff discovered new abilities in the facility’s residents, users temporarily paused their fixed roles as caregivers and care recipients [19,41]. If we hope to improve the well-being of older people in care settings, we need to design a method that transfers the workshop effects to older adults’ daily care [20,21,45]. Facilitators and care staff should communicate and work together to balance supporting and observing older participants and plan fun activities to incorporate into daily care as preparation for follow-up of the workshop [19,35] (pp. 59–71). In that way, the program will be tailored more precisely to each site and will enrich daily life and recreation time.

The present study has several limitations. First, field observations were limited to workshop implementation. Situating the effects of this activity in a broader context of people living in care facilities would require long-term observations. Second, the well-being scale Ikigai-9 was not developed primarily for use with older participants who have cognitive decline [34]; however, more than half of our participants had some level of cognitive decline. Furthermore, our sample size might not have been large enough to demonstrate the preworkshop and postworkshop changes in participants’ well-being using the Ikigai-9 scale. Older participants’ comments gathered using the postworkshop questionnaire might have been too short to provide an understanding of their individual experiences. Future research should explore more deeply how older participants, including those with dementia, feel about this activity. Third, because the larger purpose of the research project was to develop a manual for intergenerational theater workshops in care settings, the two cases had major differences in environment, which dispersed the analysis focus. If the implementation environment could be aligned (e.g., attributes of intergenerational participants, level of facility staff involvement), we could examine the relationship between the practice and outcomes in a more focused approach. Fourth, the theater workshop in this research study used an improvisational approach. However, other styles of theater, such as using reminiscence [15,17,20], may have different effects on the relationships between participants. Despite those limitations, intergenerational theater was introduced in care facilities as a new initiative in Japan and exhibited potential benefits. The evaluation of practices in different settings can be used to improve the program in the future. We advocate the value of the intergenerational theater in care settings as an approach that can bring a cultural change to routine care at facilities [20] and enhance the relationships among older people, caregivers, and community residents.

## 5. Conclusions

This research examined the novel intergenerational theater activities in Japanese older adults’ care settings. Our intergenerational theater workshops presented unique experiences for older adults and younger participants. Older participants demonstrated active responses and surprising ability when facility staff members discovered their remaining skills, and younger participants built a stronger relationship with older participants. By comparing two different settings, it is suggested including children in the activities and engaging facility staff members in workshop preparation, and follow-up activities could improve the effectiveness of the program. Future research should explore more deeply how older participants, including those with dementia, are experiencing this activity and how they benefit from it. In addition, we need to seek methods to make the activity more impactful for the older adults’ well-being by integrating it into everyday life in the facility.

## Figures and Tables

**Table 1:** Theater Together Project (1 h per session).

Session	Activity Contents
1	Creating a nickname and a name tag for each participant Self-introduction and communication games Brief introduction of the play’s storyline
2	Greetings and communication games Casting proposal Play practices led by facilitators and creation of a story outline
3	Greetings and communication games Play practice (whole group) and with small, break-out groups
4	Greetings and communication games Play rehearsal (run through) Performance in front of the audience Sharing impressions

**Table 2:** Characteristics of the older participants.

	Site 1	Site 2	Reference
Number of older participants signed up for the workshop			
	11	16	
Average age			
	86.4 ± 3.3 (82–91)	87.0 ± 8.0 (70–98)	
Gender			
Male	1	4	
Female	10	12	
Day care use or resident			
Day care	11	8	
Resident		8	
Certification of needed support/long-term care need *			
Independent		2	
Support level 1			Needs some support for task-based activities in daily life
Support level 2	1		
Care level 1	2	5	Difficulty in performing essential daily life activities by himself/herself
Care level 2	6	4	
Care level 3	1	2	Requires almost constant care
Care level 4	1	3	Faces difficulty living without constant care
Care level 5			Requires almost constant care to live
Level of independent living based on the physical ability *			
Independent	6	4	
A1	2	4	Needs support to go outside
A2	2	6	
B1	1		Requires assistance for mobility in a house
B2		2	
C2			Mostly staying in bed
C3			
Level of independent living based on the cognitive ability *			
Independent	1	2	
I	1		Mildly demented but can mostly live independently
II a		8	Can live independently with someone’s support
II b	7	5	
III a	2	1	Difficulty in behavior and communication, which necessitates care
III b			
IV			Frequent issues in behavior and need for constant care
M			Needs specialized medical care

* The certified level of care needs, physical ability, and cognitive ability are based on the governmental system of long-term care insurance.

**Table 3:** The results of the Ikigai-9 scale of older participants’ Wilcoxon signed-rank test of the pre–post score (*n* = 18) and the implementation environment of each site.

		Site 1			Site 2		
	Number of Respondents Who Answered Both in Pre- and Post-Workshop	*n* = 10			*n* = 8		
Ikigai-9 scale of older participants: Wilcoxon signed-rank test of pre- and post-workshop	Average age	86.7 ± 3.3 (82–91)			87.6 ± 9.5 (70–98)		
	Certified care level	Support level 2–Care level 4			Independent–Care level 4		
	Items: Ikigai-9 Scale	Pre *	Post *	*p* **	Pre *	Post *	*p* **
	Total score (9–45 point)	30.00 (7.45)	33.22 (5.72)	0.042	31.25 (9.62)	34.38 (8.45)	0.108
	I. Emotions towards one’s life (3–15 point)	9.30 (3.47)	13.00 (1.56)	0.020	11.25 (3.77)	12.88 (2.48)	0.168
	(1) I often feel that I am happy. (1–5 point)	3.30 (1.25)	4.60 (0.52)	0.017	3.75 (1.58)	4.25 (1.04)	0.461
	(4) I have room in my mind. (1–5 point)	2.80 (1.40)	4.00 (1.05)	0.066	4.00 (1.07)	4.38 (0.92)	0.083
	(7) My life is mentally rich and fulfilled. (1–5 point)	3.20 (1.32)	4.40 (0.52)	0.027	3.50 (1.51)	4.25 (1.04)	0.167
	II. Attitudes towards one’s future (3–15 point)	11.44 (2.46)	11.44 (2.01)	1.000	10.50 (3.46)	10.88 (3.44)	0.590
	(2) I would like to learn something new or start something. (1–5 point)	3.89 (1.27)	3.56 (1.01)	0.438	3.38 (1.51)	3.38 (1.51)	1.000
	(5) I am interested in many things. (1–5 point)	3.40 (1.43)	4.60 (0.70)	0.016	3.88 (1.13)	4.25 (1.04)	0.083
	(8) I would like to develop myself. (1–5 point)	4.30 (0.48)	3.50 (1.65)	0.102	3.25 (1.28)	3.25 (1.49)	0.861
	III. The acknowledgement of one’s existence (3–15 point)	9.78 (3.23)	8.78 (3.15)	0.202	9.50 (3.12)	10.63 (3.85)	0.320
	(3) I feel that I am contributing to someone or the society. (1–5 point)	3.60 (1.51)	3.10 (1.37)	0.096	2.75 (1.49)	3.63 (1.30)	0.168
	(6) I think that my existence is needed by something or someone. (1–5 point)	3.60 (1.51)	3.30 (1.16)	0.579	3.25 (1.28)	3.75 (1.58)	0.194
	(9) I believe that I have some impact on someone. (1–5 point)	2.89 (1.17)	2.67 (1.32)	0.516	3.50 (1.14)	3.25 (1.28)	0.683
Qualitative data of implementation environment	Younger participants	3 to 4 Adults (30s and 40s) who responded through workshop providers’ social networks, and 3 to 4 children (3 to 11 years old).			3 to 11 Neighborhood volunteers who belongs to a local amateur theater group and a public consumer center (40 s to 70 s).		
	Facility staff	The facility manager remained in the room, other staff came in and out, most of them saw the final performance.			2 to 4 staff attended the workshop site and enthusiastically supported the participation of the facility users.		
	Relationship of the facility and the workshop provider	Second time to conduct theater workshops. The facility is in the same city as the workshop providers’ base.			First time to conduct theater workshops. The facility is in the different region to the workshop providers’ base.		
Meta-inference	Implications for improving the effectiveness of the activity, obtained from a comparison of the two sites.	-May be more challenging to satisfy everyone when the participants’ cognitive and physical conditions are more varied. - Continuity of the activity could create relax atmosphere among the participants, and for the facilitator’s ability of program arrangement. - Children’s participation may have more direct impact on present well-being for older participants. The benefit of adults’ participants may take a little longer to find an impact. - The facility staff may need to explore a balance between supporting and waiting to give older participants some space to display their ability.					

* Average ± SD, ** Wilcoxon Signed Rank Test, *p* < 0.05.

## Data Availability

Due to the nature of this research, participants of this study did not agree for their data to be shared publicly, so supporting data is not available.
